# Ki-Cook: clustering multimodal cooking representations through knowledge-infused learning

**DOI:** 10.3389/fdata.2023.1200840

**Published:** 2023-07-24

**Authors:** Revathy Venkataramanan, Swati Padhee, Saini Rohan Rao, Ronak Kaoshik, Anirudh Sundara Rajan, Amit Sheth

**Affiliations:** ^1^Department of Computer Science, Artificial Intelligence Research Institute, University of South Carolina, Columbia, SC, United States; ^2^Department of Computer Science, Wright State University, Dayton, OH, United States; ^3^Department of Computational Science and Engineering, Technical University of Munich, Munich, Germany; ^4^Department of Computer Science, University of California, Los Angeles, Los Angeles, CA, United States; ^5^Department of Computer Science, University of Wisconsin Madison, Madison, WI, United States

**Keywords:** cooking process modeling, cross-modal retrieval, ingredient prediction, knowledge-infused learning, multimodal learning, representation learning, clustering

## Abstract

Cross-modal recipe retrieval has gained prominence due to its ability to retrieve a text representation given an image representation and vice versa. Clustering these recipe representations based on similarity is essential to retrieve relevant information about unknown food images. Existing studies cluster similar recipe representations in the latent space based on class names. Due to inter-class similarity and intraclass variation, associating a recipe with a class name does not provide sufficient knowledge about recipes to determine similarity. However, recipe title, ingredients, and cooking actions provide detailed knowledge about recipes and are a better determinant of similar recipes. In this study, we utilized this additional knowledge of recipes, such as ingredients and recipe title, to identify similar recipes, emphasizing attention especially on rare ingredients. To incorporate this knowledge, we propose a knowledge-infused multimodal cooking representation learning network, Ki-Cook, built on the procedural attribute of the cooking process. To the best of our knowledge, this is the first study to adopt a comprehensive recipe similarity determinant to identify and cluster similar recipe representations. The proposed network also incorporates ingredient images to learn multimodal cooking representation. Since the motivation for clustering similar recipes is to retrieve relevant information for an unknown food image, we evaluated the ingredient retrieval task. We performed an empirical analysis to establish that our proposed model improves the Coverage of Ground Truth by 12% and the Intersection Over Union by 10% compared to the baseline models. On average, the representations learned by our model contain an additional 15.33% of rare ingredients compared to the baseline models. Owing to this difference, our qualitative evaluation shows a 39% improvement in clustering similar recipes in the latent space compared to the baseline models, with an inter-annotator agreement of the Fleiss kappa score of 0.35.

## 1. Introduction

Over the recent few years, people have become more aware of their food choices due to its impact on their health and chronic diseases. Consequently, the usage of dietary assessment systems has increased, most of which predict calorie information from food images. Various such dietary assessment systems have shown promising results in nudging users toward healthy eating habits (Jospe et al., 2015; Wang et al., 2016). Furthermore, recent studies (Salvador et al., 2017, 2021; Carvalho et al., 2018; Wang et al., 2019, 2021; Zhu et al., 2019; Fu et al., 2020; Zan et al., 2020; Guerrero et al., 2021; Papadopoulos et al., 2022) have established the benefits of cross-modal representation learning in which the relevant information such as ingredients and cooking methods can be determined from a food image using an image-to-recipe retrieval task.

**Figure 1:**
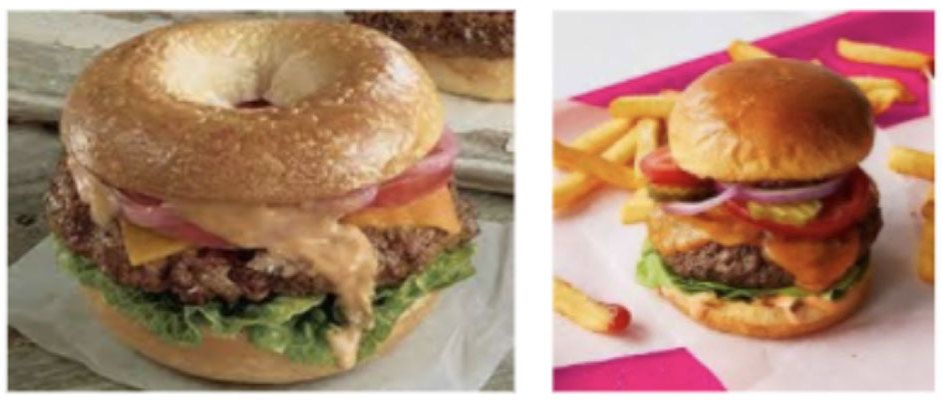
The image on the right is a burger. The image on the left could be perceived as a type of burger made with bagel buns or a bagel with stuffed vegetables. Based on our interpretation of the food item on the left, the class name can be a bagel or a burger. This is also an example of inter-class similarity where recipes from different classes can be similar (bagel or burger for the image on the left). In general, burgers also experience intraclass variation, that is, multiple sub-categories of burger (hamburger, beef burger, and so on).

Existing models (Guerrero et al., 2021; Salvador et al., 2021; Papadopoulos et al., 2022) have achieved state-of-the-art results in retrieving text representation^1^, given a food image representation and vice versa in the presence of their respective *ground truth* representation^2^. However, for an unknown food image, the *nearest* text representation must be retrieved to obtain cooking instructions and ingredients as the ground truth will not be known. For this reason, the nearest text embedding should be from a recipe^3^ similar to the recipe of the unknown food image. Hence, clustering learned representations of similar recipes and distinguishing learned representations of different recipes in the latent space are essential. Most of the existing studies (Salvador et al., 2017; Carvalho et al., 2018; Wang et al., 2021) have clustered recipes in the latent space based on class names. However, a recipe may not be associated with a single class label, as shown in Figure 1. Figure 1 also illustrates an example of the prevalent problems in the food domain known as inter-class variations, where recipes from different classes are similar, and intraclass variations, where recipes from the same class are different (sub-categories of a class). The burger buns and bagel buns have a difference of ~100 calories (Nutritionix, 2023), and hence, positioning the recipes in the right cluster is essential. Several studies (George and Floerkemeier, 2014; Silva et al., 2020; Zhao et al., 2020) have explored food classification as a multi-label problem that will require extensive manual annotations of food class labels. This problem requires additional knowledge about the recipes besides class names to identify similar recipes.

Two recipes are said to be similar if they share the same title, same set of ingredients, and same cooking actions. The recipe titles, ingredients, and cooking methods provide detailed knowledge about recipes. Rare ingredients and cooking methods play a particularly vital role in determining similar recipes. For example, two recipes can be told apart based on a rare ingredient, such as an eggplant, but not based on common ingredients, such as salt or oil, which are present in almost all recipes. Furthermore, common ingredients such as salt, sugar, and oil are not sufficient for analyzing a given recipe in the context of an allergy, a particular diet, or a health condition.

In this work, we propose a novel recipe similarity determinant that utilizes additional knowledge about recipes such as titles and ingredients, with emphasis on rarely-used ingredients. To incorporate such knowledge, we propose a knowledge-infused learning network, Ki-Cook, that clusters multimodal representations of recipes based on this similarity determinant. Knowledge-infused learning is an approach to integrate knowledge into training machine and deep learning models to improve their predictive capabilities (Valiant, 2006; Sheth et al., 2019; Garcez and Lamb, 2020). As this approach uses additional knowledge to identify similar recipes, it resolves the problem of intraclass variation and inter-class similarity described in Figure 1, introduced due to class names. To the best of our knowledge, this is the first study to utilize comprehensive knowledge about the recipes to identify similar recipe representations and cluster them in the latent space through a knowledge-infused learning approach.

Ki-Cook models the procedural attribute of the cooking process and incorporates a visual representation of ingredients to learn multimodal cooking representation. The procedural attribute of the cooking process, modeled as a sequence of states, captures the cooking actions performed with each ingredient. For this study, we also extended the largest multimodal recipe dataset Recipe1M (Salvador et al., 2017) to include 500 images per ingredient category, constituting 8 million ingredient images, and utilized them for representation learning. This is the first study to include images of 16 K ingredient categories to learn multimodal cooking representation. We plan to release our dataset to promote further research.

To cluster learned representations of similar recipes in the latent space, we have summarized the specific contributions of this article as follows: (i) a comprehensive similarity calculation approach that utilizes additional knowledge about recipes such as title and ingredients, adding attention to rarely used ingredients (ii) procedural modeling of the cooking process to learn cooking representations, (iii) incorporating visual information of ingredients in multi-modal cooking representation learning, and (iv) evaluate on ingredient retrieval task to demonstrate the ability of our similarity determinant to cluster similar recipes to retrieve relevant information for an unknown food image.

Furthermore, we also performed qualitative evaluations to analyze the clustering of similar recipes in the latent space compared to baseline models. Through experiments, we have demonstrated that our proposed knowledge-infused multimodal representation learning network identifies similar recipes better than baseline models and clusters them. Compared to baseline models, the ingredients retrieved by our learned representations are more relevant to unknown food images.

## 2. Related works

The recent growth of dietary assessment systems has led to a variety of research in food computation models varying from food image classification to food perception (Min et al., 2019). Cross-modal recipe retrieval learning is a widely researched area as the representations can be utilized for various downstream tasks.

### 2.1. Learning cross-modal recipe representations

Salvador et al. (2017) proposed a deep learning network for cross-modal recipe retrieval using the Recipe1M dataset. Building on this research, Carvalho et al. (2018) used a triplet loss-based objective function to improve the retrieval results. Zhu et al. (2019) designed a GAN-based architecture for recipe representation learning. Authors of various studies (Wang et al., 2019, 2021; Fu et al., 2020; Zan et al., 2020) have proposed the attention mechanism-based architecture to enhance the cross-modal alignment in the latent space. Salvador et al. (2021) and Guerrero et al. (2021) used hierarchical transformer-based architecture for cross-modal recipe retrieval. Papadopoulos et al. (2022) generated program representation for the cooking procedure. Various existing works (Salvador et al., 2017; Carvalho et al., 2018; Zhu et al., 2019) clustered representations of similar recipes in the latent space based on class names. Using a class name as a recipe similarity determinant would not be sufficient as recipes may not be associated with a class name (Figure 1). The existing works focus on cross-modal retrieval in the presence of ground truth representation. However, in a real-world scenario, the ground truth cooking representation is not known for an unknown food image. For this reason, our work focuses on clustering similar recipes in the latent space using additional knowledge about the recipes besides class names. Further, we evaluate on ingredient retrieval from the learned representations in the absence of ground truth representations.

### 2.2. Knowledge-infused learning

With promising results, knowledge-infused learning approaches (Dash et al., 2022) are making advances in various research fields such as autonomous driving (Wickramarachchi et al., 2021), conversational agents (Gaur et al., 2021), medical imaging (Tan et al., 2019; Zhang et al., 2020), and generative models (Lan et al., 2019). Using Recipe1M dataset, various knowledge graphs for different purposes have been introduced (Haussmann et al., 2019; Chen et al., 2021; Seneviratne et al., 2021; Shirai et al., 2021). RECIPTOR (Li and Zaki, 2020) used FoodKG (Haussmann et al., 2019) to mine triplets for their objective function and evaluated the representations for the cuisine prediction task. However, the infusion of domain knowledge into training the deep learning models for cooking representations remains unexplored. In this study, we have explored the use of domain knowledge to identify similar recipes and cluster them to improve relevant information retrieval of an unknown food image.

### 2.3. Ingredient analysis

Identifying ingredients from food images is challenging as their visibility and shape are transformed due to the cooking process. Chen and Ngo (2016) and (Chen et al., 2020) employed a multi-task multi-relational GCN for zero-shot ingredient recognition. However, detecting invisble ingredients is not possible through this approach. Salvador et al. (2019) focused on generating cooking instructions and ingredients from food images using generative models. Li et al. (2019) proposed techniques for predicting the amount of relative food ingredients from food images using the Recipe1M dataset, only focusing on the top 4 k frequent ingredients that were further reduced to 1.4 k ingredient categories. Li et al. (2021) proposed a picture-to-amount deep learning architecture model called PITA to predict 1.4 K ingredients and estimate the relative amount of ingredients using cross-modal representations. The approach proposed by PITA (Li et al., 2019) can predict the ingredients that are invisible and deformed. The study attempts to predict only the most frequently used ingredients. However, frequently occurring ingredients such as salt, sugar, and oil do not provide sufficient information to analyze the recipe in the context of an allergy, diet, or health condition. In our work, we investigate the retrieval of visible, invisible, and deformed ingredients that may be used frequently or rarely for an unknown food image. We also illustrate the significance of rarely-used ingredients in enhancing the clustering learned representation of similar recipes, thereby improving ingredient retrieval for unknown food images.

## 3. Methodology

### 3.1. Definitions and notations

The network aims to cluster the representations of food images and the respective cooking procedures of similar recipes in the latent space. To achieve this clustering, the common latent space is learned for food images and cooking procedures where they are clustered. Formally, a given recipe *r* = {*D, S*}, where *D* is a dish image and *S* is a sequence of states ranging from *s*_1_ to *s*_*n*_, where *n* is the final state of the recipe. The sequence of states can be viewed as a sequence of actions performed on the ingredients to complete a recipe. The dish image *D* corresponds to the appearance of the food image obtained after completing the cooking procedure's final step *s*_*n*_. Each state *s*_*i*_ = {*c*_*i*_, *t*_*i*_, *v*_*i*_}, where *c*_*i*_ corresponds to the cooking instruction in the text, *t*_*i*_ corresponds to the ingredient name and volume in the text, and *v*_*i*_ corresponds to the ingredient image present in the cooking instruction. Henceforth, the ingredient name and volume in text *t*_*i*_ would be referred to as ingredient text for brevity.

### 3.2. Data collection and pre-processing

#### 3.2.1. Dataset extension

For this study, we extended the Recipe1M dataset (Salvador et al., 2017), which consists of more than one million recipes, to include ingredient images. The Recipe1M dataset consists of dish images, recipe title, ingredient text, and instruction text for a given recipe. The dataset has 9 million ingredients, meaningfully reduced to 16 K ingredients by Salvador et al. (2017). For the 16 k ingredients, we used the ingredient name as the query and extracted the top-500 results from Google Images, which resulted in 8 million ingredient images. For the scope of this research, we did not filter the images based on their quality or relevance and regard them as noise in the training data. Instead, we have presented the quality assessment of ingredient images in Section 4.4.

#### 3.2.2. Instruction pre-processing

Our proposed approach models the cooking procedure as a sequence of states, therefore, we processed the cooking instructions to have one ingredient per instruction. We employed the spaCy NLP parser (Honnibal and Montani, 2017) to extract the noun phrases from a given cooking instruction. Each recipe in the Recipe1M dataset consists of a set of preprocessed ingredients ING = {*ing*_1_, *ing*_2_, .., *ing*_*n*_} in a textual format. We observed variations in ingredient names present in the list of noun phrases [*t*] extracted by spaCy compared to the ingredient names present in the ING set. For example, *Philadelphia cream cheese* in the ingredient set ING is present as *cream cheese* in the cooking instruction. Hence, to address this challenge, we computed the Intersection Over Union (IOU) of word tokens over each item in the extracted noun phrases [*t*] with each ingredient in the set ING. For a noun phrase present in [*t*], we considered the ingredient with the highest IOU in the set ING as a match. Then, we used the ingredients from the list of noun phrases [*t*] as an end-of-sentence marker to split the cooking instruction.

### 3.3. Model architecture

**Figure 2:**
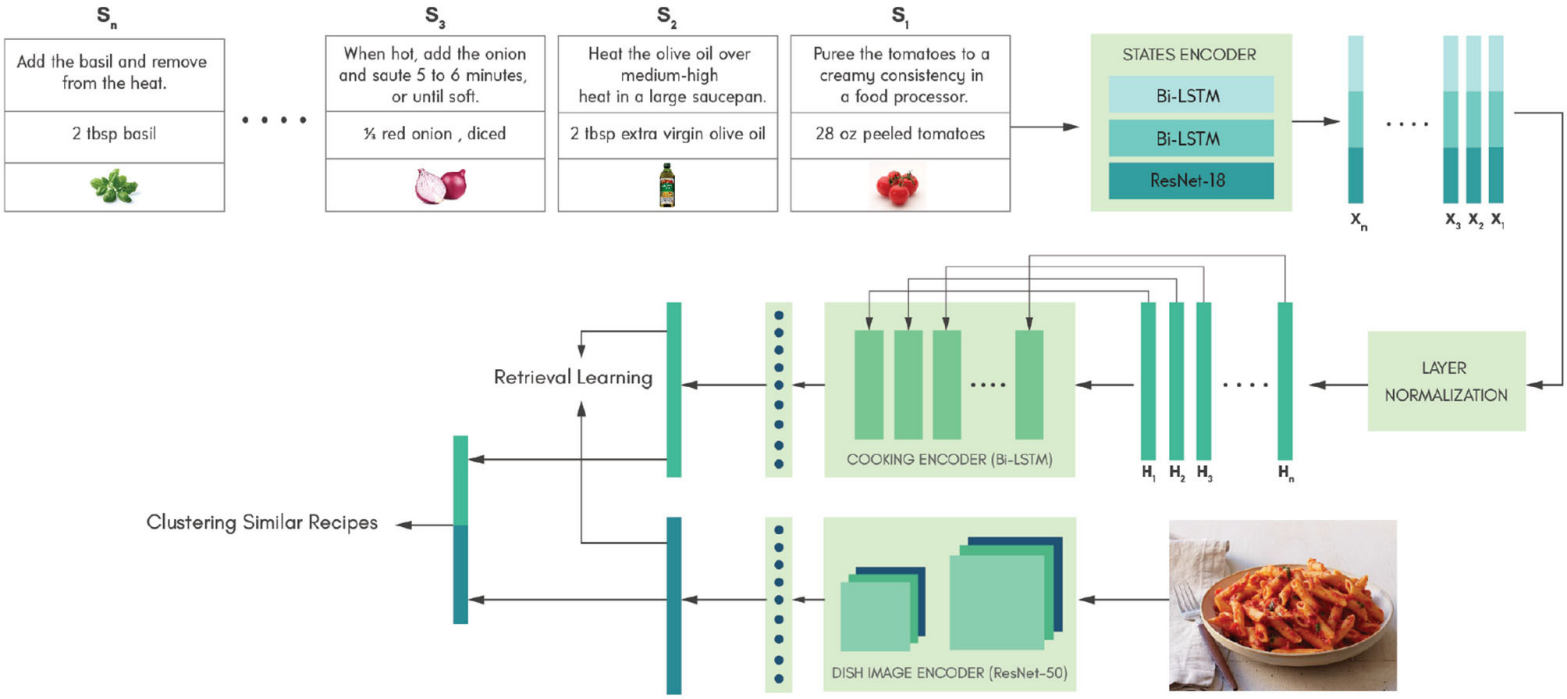
The overall network architecture of the proposed approach is illustrated in this figure with an example recipe, red sauce pasta. The state encoder takes each state of a recipe as the input in sequential order to produce a learned representation for each state. The cooking encoder takes the learned state representations in sequential order to generate a final learned representation for the cooking procedure. The learned cooking representation and dish image representation are clustered in the latent space based on the knowledge infused through the similarity determinant.

In this section, we have described our proposed model architecture shown in Figure 2. To demonstrate that using the same models used by Salvador et al. (2017) but modeling procedural attributes of the cooking process and infusing knowledge can improve relevant information retrieval for an unknown food image, we only used the same model as that used by Salvador et al. (2017) and evaluated our model against theirs. The proposed model architecture comprises three primary encoders, i.e., a states encoder, a cooking encoder, and a dish image encoder, which have been discussed below.

#### 3.3.1. States encoder

**Figure 3:**
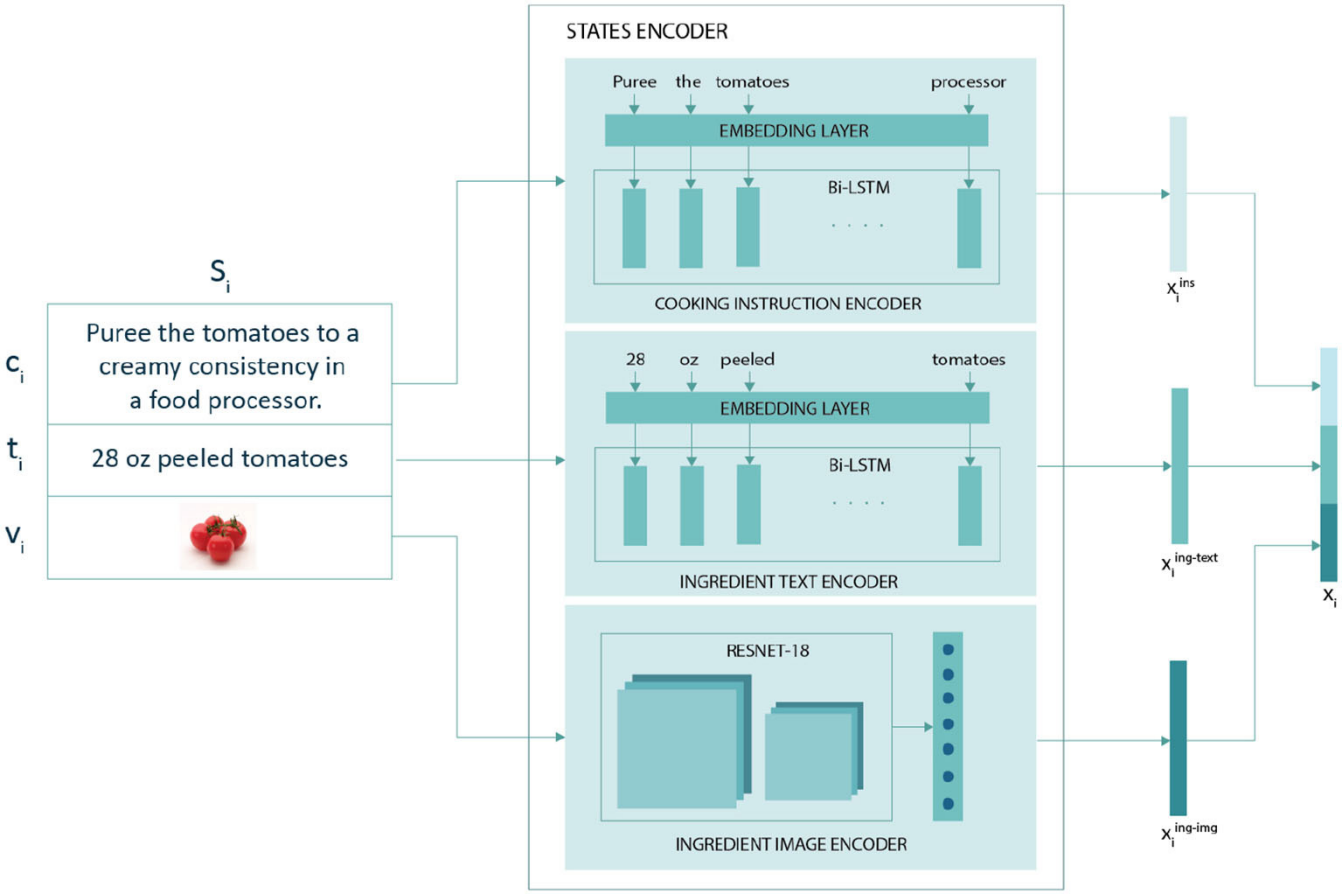
The state *S*_*i*_={*c*_*i*_, *t*_*i*_, *v*_*i*_} where *c*_*i*_ corresponds to the *i*th cooking instruction in the text, *t*_*i*_ and *v*_*i*_ correspond to the ingredient text and ingredient image, respectively, present in the *i*th cooking instruction. The three learned representations $x_{i}^{ins}$, $x_{i}^{ing-text}$, and $x_{i}^{ing-img}$ are concatenated to form the state representation *x*_*i*_ for *i*th state.

The states encoder generates representations for each state in the recipe (Figure 3). Each state in the recipe consists of a cooking instruction, the ingredient name and volume, and an ingredient image to capture actions performed on an ingredient at a given time step. A recipe consists of n states from *s*_1_ to *s*_*n*_ and its corresponding state representation *x*_1_ to *x*_*n*_ is generated by the states encoder. The representation of the *i*th state *x*_*i*_ was obtained by concatenating *i*th representations of cooking instruction $x_{i}^{ins}$, ingredient text $x_{i}^{ing-text}$, and ingredient image $x_{i}^{ing-img}$, as described in Equation (refeq:concatenation)


(1) $$\begin{array}{l} x_{i}=\left[x_{i}^{ins},x_{i}^{ing-text},x_{i}^{ing-img}\right]\;, \end{array}$$

The states encoder consists of a cooking instruction encoder, an ingredient text encoder, and an ingredient image encoder, as discussed below.

##### 3.3.1.1. Cooking instruction encoder

The cooking instruction encoder generates a representation for a given cooking instruction. It consists of a learnable embedding layer, which is followed by a bidirectional long short-term memory networks (LSTM). The learnable embedding layer was set to 300 dimensions and generates encoding for words. The bidirectional LSTM utilized the learned word embeddings to generate a representation $x_{i}^{ins}$ for the cooking instruction *c*_*i*_ in state *s*_*i*_. The hidden LSTM layer was set to 300 dimensions. We concatenated the output from the last hidden layer of both directions to get the representation for the cooking instruction.

##### 3.3.1.2. Ingredient text encoder

Curating a dataset for all forms of an ingredient, such as diced and pureed tomatoes, is a tedious task. Thus, we used the ingredient text that represents the form and volume of the ingredient along with the ingredient image. Similar to the cooking instruction encoder, the ingredient text encoder consists of a learnable embedding layer and bi-directional LSTM to obtain the representation $x_{i}^{ing-text}$ for the ingredient text *t*_*i*_ in state *s*_*i*_. The embedding layer and the bidirectional LSTM of the cooking encoder and ingredient text encoder share their weights.

##### 3.3.1.3. Ingredient image encoder

The final dish image of the recipe resulted from the ingredients changing appearance due to a sequence of cooking actions. Hence, we incorporated ingredient images to acquire a visual representation of ingredients. We use ResNet-18 to encode the ingredient images, and the final softmax layer was removed. The output from the last average pooling layer was fed to a fully connected layer to generate the representation $x_{i}^{ing-img}$ of 512 dimensions for the ingredient image *v*_*i*_ present in state *s*_*i*_.

#### 3.3.2. Cooking encoder

The cooking encoder consists of a bidirectional LSTM to capture the global temporal dependency of the cooking procedure. It was established that normalizing hidden layers can stabilize the training process. Hence, similar to Wang et al. (2021), we introduced a normalization layer described by Ba et al. (2016) to normalize the state representations [*x*_1_, *x*_2_,..., *x*_*n*_] before passing it to the bidirectional LSTM. The LSTM takes a sequence of normalized state representations [*H*_1_, *H*_2_,..., *H*_*n*_] as its input and generates a representation for the cooking procedure. Each state representation is a 1,12 dimensional vector. Correspondingly, the hidden layer of bidirectional LSTM was set to 1,712 dimensions. Finally, we concatenated the output from the last hidden layer of both directions and passed it to a fully connected layer of 1,024 dimensions to obtain the final cooking representation.

#### 3.3.3. Dish image encoder

We adopted the ResNet-50 model to extract the visual features from dish images *D*. We removed the final softmax layer to obtain a representation of 2,048 dimension from the last average pooling layer. The learned representation was then passed to a fully connected layer of 1,024 dimensions to obtain the final dish image representation.

### 3.4. Objective function

Inspired by prior studies from Carvalho et al. (2018); Zan et al. (2020); Wang et al. (2021), which obtained promising results, we used triplet loss as an objective function to learn the common latent space for dish image and multimodal cooking representations. For the proposed model, we used multiple negative samples and one positive sample mined from a given batch. The triplet loss for a given data sample was calculated as described in Equation (2).


(2) $$\begin{array}{l} l_{triplet}=\overset{5}{\underset{k=1}{\sum }}\left[d\left(I_{a},K_{p}\right)-d\left(I_{a},K_{n,k}\right)\right]+\overset{5}{\underset{k=1}{\sum }}\left[d\left(K_{a},I_{p}\right)-d\left(K_{a},I_{n,k}\right)\right]\;, \end{array}$$

where *a, p*, and *n* represent the anchor, positive and negative samples; *k* represents the number of negative samples; *K* is the cooking representation; *I* is the dish image representation; and α is the margin parameter of triplet loss (Balntas et al., 2016).

### 3.5. Recipe similarity determinant

In this section, we have discussed our recipe similarity determinant that utilizes titles and ingredients of a recipe to compute a semantic similarity score to cluster similar recipe representations in the latent space. We plan to incorporate cooking methods in the similarity determinant in the future. The semantic similarity score in Equation (3) provides a degree of similarity between any two given recipe pairs (*r*_*i*_, *r*_*j*_) and we computed the score as


(3) $$\text{Φ}(r_{i},r_{j})=\frac{\sum_{i=1}^{n}w_{i}\times x}{n}+\frac{\sum_{i=1}^{m}\left(1/f_{i}\right)\times x}{m}\;,$$

where *n* is the sum of words in the titles of *r*_*i*_ and *r*_*j*_ after removing stop words; *m* is the sum of ingredients present in *r*_*i*_ and *r*_*j*_; *w*_*i*_ is the weight of each word in the title; *f*_*i*_ is the frequency of each ingredient computed over the recipes in the training, testing, and validation datasets; and *x* is 1 if the word or ingredient is present in both the recipes but 0 otherwise. The inverse frequency of ingredients in Equation (3) adds attention to the rarely used ingredients. The weight *w*_*i*_ is 1 for any word in the title and 2 if it is a class label such as pasta, burger, and so on. We utilized the class labels published by Salvador et al. (2017). We empirically chose the weight for words present in the class label and assigned weights to the class labels hypothesizing that the recipes under a given class should be closer than two similar recipes of different classes. The evaluations are presented both with and without adding weights for class weights.

#### 3.5.1. Knowledge infusion

Using the semantic similarity score, we computed semantic similarity loss to cluster recipes in the latent space based on their similarities instead of clustering based on just class names. We concatenated the dish image representation *I* and cooking representation *K* to form 2,048 dimensional representation, called recipe, representation *e*_*i*_ = [*K*_*i*_, *I*_*i*_], where i denotes the ith representation in the batch. For a given data sample in a batch, we calculated the semantic similarity loss as


(4) $$l_{sem}=\sum_{j=1}^{N-1}|\left(\frac{\text{Φ}(r_{i},r_{j,j\neq i})-\mu_{\text{Φ}}}{\sigma_{\text{Φ}}}\right)-\left(\frac{\cos(e_{i},e_{j,j\neq i})-\mu_{\cos}}{\sigma_{\cos}}\right)|\;,$$

where *N* is the batch size; μ_Φ_ and σ_Φ_ are the mean and standard deviations of the semantic similarity scores; and μ_cos_ and σ_cos_ are the mean and standard deviations of the cosine similarity scores. Equation (4) enforces the distribution of cosine similarity scores to follow the distribution of semantic similarity scores. As the cosine similarity scores followed the distribution of the semantic similarity scores, the learned recipe representations can be clustered in the latent space based on their similarities computed using the semantic similarity scores. We calculated the total loss for a given data sample as


(5) $$\begin{array}{l} Loss=l_{triplet}+\text{λ}l_{sem}\;, \end{array}$$

where λ is the trade-off parameter. For a given batch, we computed the loss for each data sample and averaged them.

## 4. Experiments

### 4.1. Dataset

The extended Recipe1M dataset (described in Section 3.2) was used for the training and evaluation of our model. Similar to the study of Salvador et al. (2017), we used 340 k unique recipes for this study. Of the 340 k recipes, 13 k have more than one ingredient but only one instruction for the entire recipe, such as “Mix all the ingredients and serve” as the states encoder takes only one ingredient per instruction. After removing the 13 k recipes, the dataset comprises 229,317 recipes for training, 49,294 for testing, and 49,075 for validation. We only included recipes with at least one dish image present.

### 4.2. Implementation details

We initialized both the ResNet (mentioned in Section 3.3) models with pretrained weights from the ImageNet dataset (Deng et al., 2009). We freezed the weights of the ingredient image encoder except for the fully connected layer at the end of ResNet-18. We initialized the rest of the network with random weights for training. We randomly sampled an image from our extended dataset for the dish images and an image from the top-100 results returned by Google Images for the ingredient images. For the states without any ingredient in the cooking instruction, we input “none” for the ingredient text and a white image for the ingredient image. We used Adam optimizer (Kingma and Ba, 2014) with a learning rate of 10^−5^. The trade-off parameter λ was set to 1, and the number of negative samples *k* in the triplet loss function was set to 5. We empirically chose the hyperparameter values. We trained the end-to-end network with a batch size of 64. We employed early stopping to prevent the model from overfitting and trained it for several epochs until it converges.

### 4.3. Evaluation protocols

As the goal of clustering was to retrieve relevant information about an unknown food image, we performed a quantitative evaluation on the ingredient retrieval task. Since this is the first study to perform ingredient retrieval from learned representations by clustering them, we created our baseline based on Salvador et al. (2017). Since Salvador et al. (2017) performed evaluations on cross-modal recipe retrieval in the presence of ground truth and not ingredient retrieval in the absence of ground truth, we performed ingredient retrieval evaluation on their model. We trained both models to the same cross-modal median retrieval rank to effectively demonstrate the difference in the quality of representations generated by both approaches. Furthermore, we performed a qualitative evaluation to analyze the clustering of recipes in the latent space based on similarity.

#### 4.3.1. Quantitative evaluation

For a given dish image representation *I*, we retrieved the *k*-nearest cooking representation *K* using cosine similarity to predict the ingredients present in the dish image. We present the results with varying *k* values to evaluate the clustering of similar recipes. In a real-world scenario, we do not have access to the ground truth cooking representation to retrieve ingredients for an unknown food image. Hence, we removed the corresponding cooking representation (ground truth) of a food image representation before finding the closest cooking representation. We used the following metrics as reported by Li et al. (2021) for quantitative results:

- **Coverage of Ground Truth (CVG):**
  (6) $$CVG=\frac{c}{\sum_{i=1}^{M}y_{i}}\;,\;c=\sum y\cap \hat{y}\;,$$
  where *y* is the ground truth ingredient set, $\hat{y}$ is the predicted ingredient set, and *M* is the total number of ingredients in the ground truth ingredient set.
- **Intersection Over Union (IOU):**
  (7) $$IOU=\frac{c}{(\sum_{i=1}^{M}y_{i}+\sum_{i=1}^{\hat{M}}\hat{y_{i}})-c}\;,$$
  where $\hat{M}$ is the total number of ingredients in the predicted set.

Since there are no established methods to evaluate the relevant information retrieval of an unknown food image using learned representations, we adapted and constructed an evaluation procedure based on the procedures introduced by Salvador et al. (2017) and Li et al. (2021). We randomly sampled a subset of 1,000 dish image and cooking representation pairs from the test set. We retrieved the *k*-nearest cooking representation using cosine similarity for each dish image representation to compute CVG and IOU. Evaluations were performed on the k-nearest cooking representation to demonstrate the efficiency of our approach to cluster similar recipes. We repeated the experiment 10 times for each *k* and reported the mean result in Tables 1, 2. We repeated the same procedure by randomly sampling 10,000 dish image and cooking representation pairs. The models used in the quantitative evaluation are as follows:

- JE: The method proposed by Salvador et al. (2017) without a semantic regularizer
- JE+SR: The method proposed by Salvador et al. (2017) with a semantic regularizer
- Ki-Cook: Our model trained only on triplet loss and without semantic similarity loss
- Ki-Cook + SSWC: Our model trained on both triplet loss and semantic similarity loss. In this model, the weight of the recipe title words that belong to the class label was set to 2, as shown in Equation (3), that is, *w*_*i*_ = 2 if the word *w*_*i*_ belongs to a class label. For example, in the recipe name *Red Sauce Pasta, Pasta* is considered the class name, as described in the study by Salvador et al. (2017).
- Ki-Cook + SSWOC: Our model trained on both triplet loss and semantic similarity loss. In this model, the weight for the recipe title words that belong to the class label was set to 1, as shown in Equation (3) (i.e., *w*_*i*_ = 1 always).

**Table 1:** Recall and IOU of the ingredient prediction task for 10,000 samples.

**Models**	***K*** **= 1**		***K*** **= 5**		***K*** **= 10**	
	**CVG (0.38)**	**IOU (0.19)**	**CVG (0.46)**	**IOU (0.27)**	**CVG (0.48)**	**IOU (0.28)**
JE	0.0685	0.0376	0.06407	0.03541	0.05628	0.03086
JE + SR	0.0717	0.0389	0.07037	0.0365	0.0702	0.0363
Ki-Cook	0.0730	0.0387	0.07135	0.0381	0.0705	0.03650
Ki-Cook + SSWC	0.07475	**0.0405**	0.0701	0.0376	0.0709	**0.0369**
Ki-Cook + SSWOC	**0.0777**	0.0393	**0.0728**	**0.0385**	**0.0719**	0.0359

As the ground truth was removed, the upper bound (maximum possible accuracy) for each run is as mentioned within the parentheses.

The bold values represent the highest CVG and IOU for a given column.

**Table 2:** Recall and IOU of the ingredient prediction task for 10,000 samples.

**Models**	***K*** **= 1**		***K*** **= 5**		***K*** **= 10**	
	**CVG (0.47)**	**IOU (0.28)**	**CVG (0.56)**	**IOU (0.38)**	**CVG (0.59)**	**IOU (0.40)**
JE	0.0679	0.0367	0.0622	0.0311	0.0591	0.0308
JE + SR	0.0763	0.0371	0.0721	0.0365	0.0695	0.0362
Ki-Cook	0.0743	0.0399	0.0736	0.0368	0.0700	0.0359
Ki-Cook + SSWC	0.0783	0.0406	0.0742	0.0377	0.0709	0.0365
Ki-Cook + SSWOC	**0.0793**	**0.0414**	**0.0747**	**0.0386**	**0.0743**	**0.0383**

As the ground truth is removed, the upper bound (maximum possible accuracy) for each run is as mentioned within the parentheses.

The bold values represent the highest CVG and IOU for a given column.

#### 4.3.2. Qualitative evaluation

For the qualitative evaluations, we used the JE + SR and Ki-Cook + SSWOC models to retrieve the respective nearest cooking representation for all dish image representations in the test set. Similar to quantitative evaluations, we excluded the corresponding cooking ground truth representation of dish images before retrieving the nearest cooking representation. We use JE + SR and Ki-Cook + SSWOC for this evaluation as they are the best performing models in quantitative analysis. Henceforth, we used the term anchor recipe to refer to the recipe whose dish image was used to retrieve the nearest cooking representation by both models.

The task was to evaluate whether the recipe of the cooking representation retrieved by JE + SR or Ki-Cook + SSWOC is similar to the anchor recipe. The annotators chose to answer neither. We randomly sampled 200 data points and distributed them among 12 annotators aged between 21 and 33 years who are graduate students from the Computer Science Department. The annotators belong to diverse ethnic groups. For each recipe, we present the recipe title and its dish image randomly sampled from the dataset to the annotators for qualitative evaluation.

## 5. Result and discussion

### 5.1. Quantitative results

From Tables 1, 2, we observed that our Ki-Cook + SSWOC model improves the CVG of the baseline models by 12% and the IOU by 10% in the ingredient retrieval task. Since the ground truth representation was removed before the evaluation, the upper bound for each evaluation is as mentioned in Tables 1, 2. Overall, the Ki-Cook + SSWOC model achieves better performance compared to other models. The results also demonstrate that not adding weights to recipe title words that belong to the class labels (Ki-Cook + SSWOC) improves the performance compared to when the weights are added (ki-Cook + SSWC). Furthermore, our knowledge-infused models (Ki-Cook + SSWC and Ki-Cook + SSWOC) performed significantly better when *k* = 5 and *k* = 10. As the k was increased, the number of similar recipes in the *k* cooking representations was reduced for the baseline model compared to our proposed approach. Similarly, when evaluated with 10,000 samples, we noted improved CVG and IOU as the number of similar recipes in the sample increased. This shows that the recipe similarity determinant is beneficial to the enhanced clustering of similar recipes in the latent space. The Ki-Cook-3 presented in Table 5 utilizes the same dataset (without ingredient images) for training as JE + SR and Ki-Cook - 1 utilizes the same dataset as JE. In both cases, Ki-Cook performs significantly better for *k* = 1. This shows that the modeling procedural attributes of the cooking process and the proposed similarity determinant improves the ingredient retrieval for an unknown food image.

Further, we performed a comparative analysis of the models on detecting rarely used ingredients and the results are presented in Table 3. The significant role played by rarely used ingredients in clustering similar recipes in the latent space is discussed in Section 5.2. As similar recipes are clustered, it enables the retrieval of a cooking representation from a recipe similar to the recipe of an unknown food image, improving the results of ingredient information retrieval of an unknown food image. The results demonstrated that all our models detected a significantly higher percentage of rarely used ingredients as compared to JE + SR (the best performing baseline model from Tables 1, 2). On average, Ki-Cook + SSWOC detects 16.7% more rarely used ingredients as compared to JE+SR. Furthermore, adding weights to class labels (Ki-Cook + SSWC) to cluster based on class names, as in existing studies, lowers the model's ability to detect rarely used ingredients, thereby diminishing its ability to cluster similar recipe representations.

**Table 3:** Percentage increase in detecting low-frequency ingredients of our model compared to the baseline (JE + SR), with *k* = 1.

**Models**	** < 1,000**	** < 2,000**	** < 3,000**	** < 4,000**	** < 5,000**
Ki-Cook	9.09%	18.27%	14.44%	11.79%	13.87%
Ki-Cook + SSWC	13.79%	14.45%	13.75%	14.02%	13.96%
Ki-Cook + SSWOC	13.79%	23.96%	21.11%	16.36%	17.41%

### 5.2. Qualitative results

**Figure 4:**
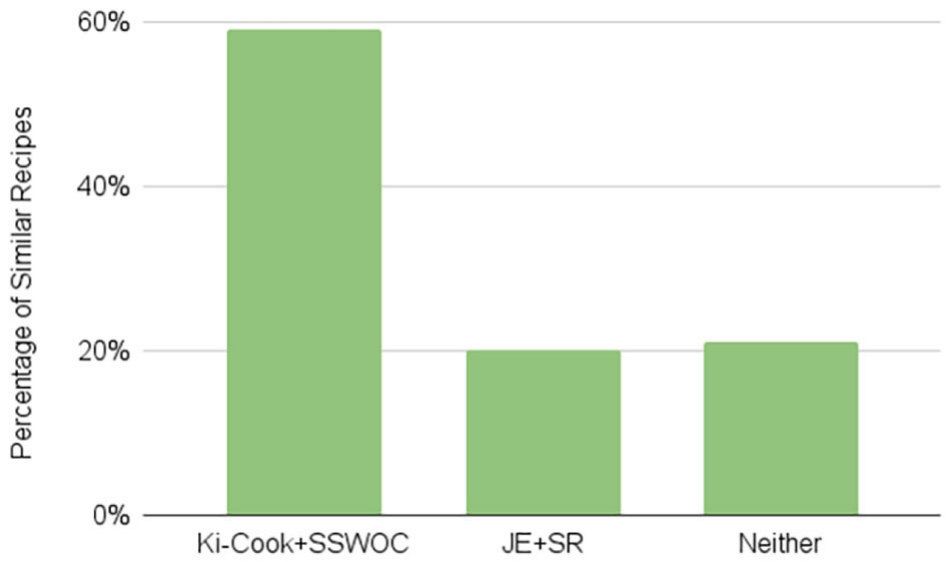
Result of qualitative evaluation. This figure consists of the percentage of similar recipes retrieved by each model. Of the 200 samples, the annotators agreed with the 0.35 inter-annotator agreement of the Fleiss kappa score that Ki-Cook + SSWOC identified similar recipes for 59% of the cases. Contrarily, JE + SR identified similar recipes for 20% of the recipes. Finally, for 21% of the recipes, neither of the models retrieved a similar recipe.

**Figure 5:**
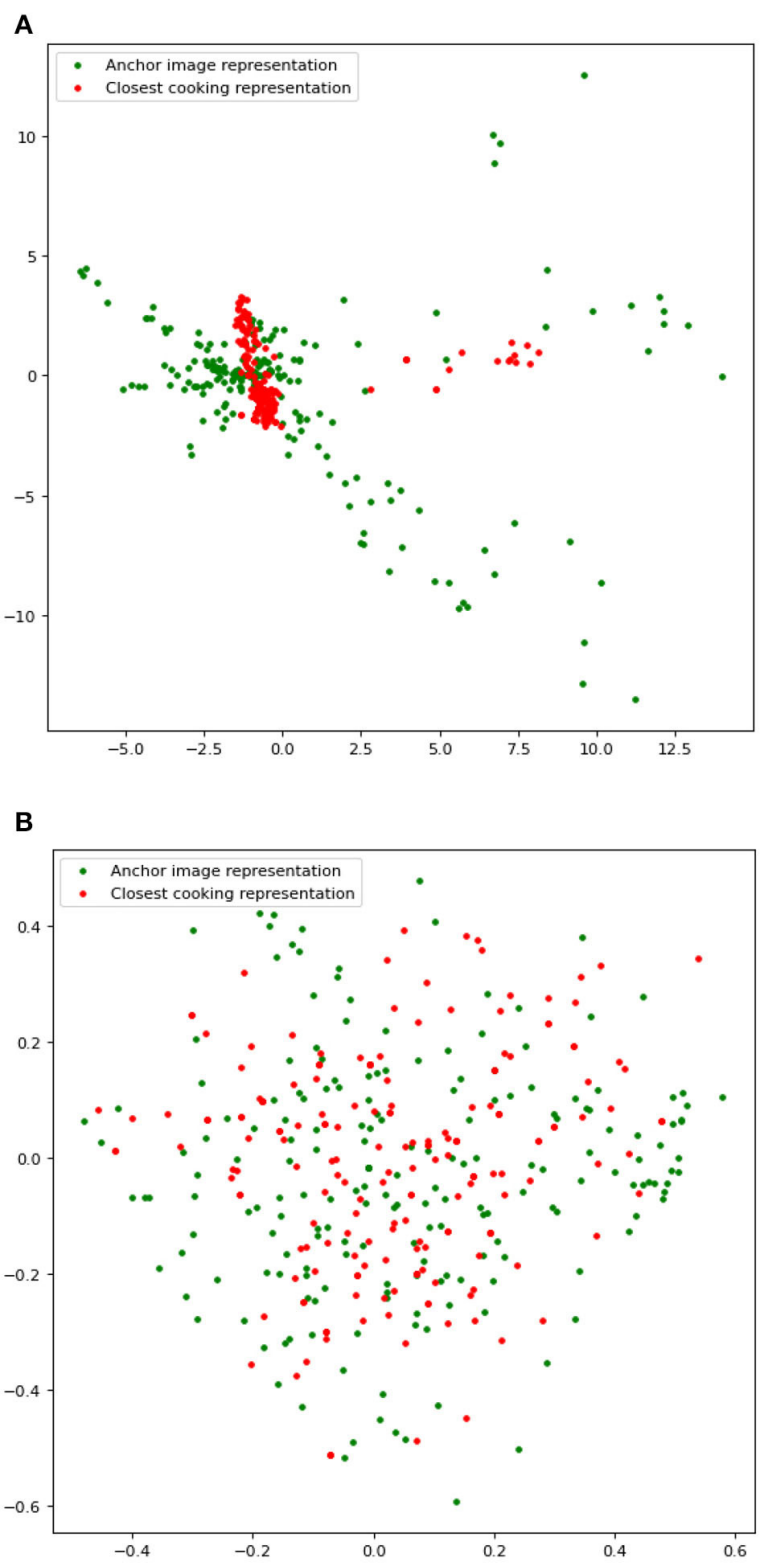
Visualization of learned representations of recipes used in our qualitative analysis. Best viewed in color. **(A)** Learned representations generated by Ki-Cook + SSWOC. The figure consists of multiple clusters clustered together in the zoomed-out view, which appear as one big cluster. **(B)** Learned representations generated by JE + SR.

In our qualitative evaluation, the annotators agreed with 0.35 inter-annotator agreement of the Fleiss kappa score that our model retrieves similar recipes for 59% of the 200 anchor recipes and that JE + SR retrieves similar recipes for 20% of the 200 anchor recipes. For 21% of the anchor recipes, neither of the models retrieved a similar recipe (Figure 4), which shows that Ki-Cook clustered the learned representation of similar recipes compared to the JE+SR model. As mentioned earlier, rarely used ingredients such as eggplant or cornstarch can determine similar or dissimilar recipes as compared to common ingredients such as salt or oil, which are used in almost all the recipes. Our model, Ki-Cook + SSWOC, predicts 15.3% more of the rarely used ingredients, as presented in Table 3. Consequently, our model demonstrated improved ability to determine similar and dissimilar recipes, clustering similar recipes in the latent space, demonstrated in Figure 5, as compared to JE + SR. This resulted in a 39% improvement in our model to return a cooking representation from a relatively similar recipe to the recipe of an unknown food image compared to JE + SR.

We illustrated the importance of rarely used ingredients in improving the clustering using the examples presented in Table 4. Specifically, we chose examples where our best performing model's (Ki-Cook + SSWOC) CVG and IOU are higher, the same, and lower than JE + SR. In all three examples, the recipe retrieved by our model is similar to the anchor recipe. In example 1, while our model's CVG is marginally better than that of JE + SR, it retrieved a similar recipe by predicting eggplant, which is relatively less frequent (13,202 occurrences) than eggs (82,217 occurrences) predicted by JE+SR. Similarly, in example 2, our model predicts shredded cheddar cheese, which has the second least frequency (15,961 occurrences) in the anchor recipe. Even though both models have the same CVG in example 2, our model retrieves the most similar recipe by identifying relatively rarely used ingredients. In example 3, the CVG of our model is less than that of JE + SR. Nonetheless, our model retrieves a similar recipe by predicting a rarely used ingredient, cornstarch (26,921 occurrences), compared to common ingredients such as brown sugar and butter predicted by JE+SR. These results showed that, irrespective of whether the CVG is higher, lower, or comparable, our Ki-Cook + SSWOC model retrieves relatively the most similar recipe compared to JE + SR owing to its ability to identify rarely used ingredients.

**Table 4:** Examples to explain the qualitative results.

**Example**	**Anchor Recipe**	**JE + SR Recipe**	**Ki-Cook + SSWOC Recipe**
1	**Fried Eggplant**	**Dilly Cheese Muffins**	**Low Carb Eggplant (Aubergine) Parmesan**
	parmesan cheese (62,979), half and half milk (44), flour (113,250), eggplant (13,202), oil (41,631),seasoned bread crumbs (1,304), and egg (82,217)	baking mix (1,040), Swiss cheese (9,744), egg (82,217), milk (112,134), vegetable oil (89,771), fresh dill (5,860), dry mustard (5,929), paper baking cups (180), and vegetable oil cooking spray (2,312)	eggplant (13,202), no—added—sugar low carb spaghetti sauce (1), parmesan cheese (62,979), mozzarella cheese (26,202), Italian seasoning (5,776), dried oregano (11,744), and dried basil (6,131)
		**Predicted:** egg	**Predicted:** parmesan cheese, eggplant
		**CVG:** 0.14	**CVG:** 0.28
2	**Cheddar and Chive Biscuits**	**Cora's World Famous Chocolate Chip Cookies!**	**Peppery Cheese and Chive Biscuits**
	baking powder (85,249), salt (303,175), sugar (224,883), shredded cheddar cheese (15,961), dried chives (269),butter (283,192), vegetable oil (89,771), and whole milk (18,482)	baking powder (85,249), sugar (224,883), all-purpose flour (131,121),butter (283,192), vanilla (41,563), eggs (206,544),baking soda (65,375), nuts (7,885), and semi-sweet chocolate chips (15,530)	baking powder (85,249), fat-free half-and-half (948), fresh coarse ground black pepper (2,745), all-purpose flour (131,121), butter (283,192), stone ground mustard (270), fresh chives (6,172), shredded cheddar cheese (15,961), and onion powder (6,399)
		**Predicted:** baking powder, butter, sugar, all-purpose flour	**Predicted:** baking powder, butter, shredded cheddar cheese, all-purpose flour
		**CVG:** 0.44	**CVG:** 0.44
3	**Sweet and Sour Chicken With Rice**	**Banoffee Pie**	**Chicken Stir Fry Oriental**
	salt (303,175), pineapple chunks (2,456), hot chicken stock (348), rice (13,752), cooked chicken (6,850), brown sugar (66,372), vinegar (8,272), dried onion flakes (743), pineapple juice (4,131), cornstarch (26,921), and butter (283,192)	bananas (19,758), water (197,699), brown sugar (66,372), caramels (2,785), lemon juice (45,714), and butter (283,192)	vegetable oil (89,771), frozen oriental - style vegetables (34), soy sauce (49,151), ground ginger (8,879), cornstarch (26,921), boneless chicken breasts (8,150), cooking sherry (420), and sugar (224,883)
		**Predicted:** brown sugar, butter	**Predicted:** cornstarch
		**CVG:** 0.181	**CVG:** 0.09

Three examples were chosen, where the CVG of our model is higher, lower, and equal to the CVG of the JE + SR model. The ingredient frequency was denoted within the parentheses next to each ingredient.

When the detected ingredients were analyzed for the 200 recipes used in the qualitative evaluation, 9.5% of the ingredients detected by our model have a frequency of <5,000, while 4.5% of the ingredients detected by JE+SR have a frequency of <5,000. Furthermore, the ingredients retrieved by our model for the unknown food image include dominant ingredients (present in the title of the recipe) such as eggplant and shredded cheddar cheese. Therefore, the rarely used ingredients played a vital role in determining similar and dissimilar recipes, thereby clustering similar recipes in the latent space. This resulted in retrieving a cooking representation from a recipe similar to the recipe of an unknown food image, improving the results of ingredient information retrieval. The retrieved ingredient images for the examples presented in Table 3 are included as Supplementary material.

The improvement in our quantitative evaluations is not as significant as the improvement in our qualitative evaluations because JE+SR achieves its CVG and IOU by predicting commonly used ingredients. It is worth noting that the top 4k ingredients with the highest frequency account for an average coverage of 95% (Li et al., 2019).

### 5.3. Ablation study

We conducted an ablation study with four versions of our model to evaluate the effectiveness of ingredient images (ING-IMG) and semantic similarity loss (SSL). The four versions are (i) Ki-Cook-1(without ING-IMG and SSL), (ii) Ki-Cook-2 (with ING-IMG and without SSL), (iii) Ki-Cook-3 (without ING-IMG and with SSL), and (iv) Ki-Cook-4 (with ING-IMG and SSL). We observed from Table 5 an 8.8% improvement in the CVG and a 3.6% improvement in the IOU for Ki-Cook-4 compared to Ki-Cook-1, which neither uses ING-IMG nor SSL. We also studied the importance of ING-IMG and SSL in isolation through Ki-Cook-2 and Ki-Cook-3. The CVG and IOU of Ki-Cook-4 are higher than that of Ki-Cook-2 and Ki-Cook-3. The results in Table 5 indicated the significance of ingredient images and semantic similarity loss in improving our proposed model's overall performance. Additionally, the Ki-Cook-1 in Table 5 utilized the same data (ingredient text, cooking instruction, and dish image) as JE and JE + SR in Table 1. Therefore, the CVG and IOU improvement of Ki-Cook-1 compared to JE and JE+SR also validates the effectiveness of procedural modeling of the cooking process.

**Table 5:** Ablation study to study the effectiveness of ING-IMG and SSL.

**Models**	**CVG**	**IOU**
Ki-Cook-1 (without ING-IMG and SSL)	0.0714	0.0379
Ki-Cook-2 (with ING-IMG and without SSL)	0.0730	0.0387
Ki-Cook-3 (without ING-IMG and with SSL)	0.0745	0.0388
Ki-Cook-4 (with ING-IMG and SSL)	0.0777	0.0393

The experiments are conducted without ground truth for 1,000 samples and *k* = 1.

### 5.4. Ingredient image analysis

We performed evaluations to assess the quality of ingredient images. While collecting ingredient images from Google Images, we saved the images in the order in which Google Images returned the results. Then, we removed non-jpeg, non-png, and corrupted files. For quantitative assessment of noise, we randomly sampled 5 images from the top-10, top-100, and top-500 images for randomly sampled 50 ingredients. We then evaluated whether the five images are relevant to the ingredient name. The assessment showed that 68% of images are relevant from the top-10, 67% are relevant from the top-100, and 54% are relevant from the top-500 images. We found that most of the noise was due to entity ambiguation, such as *apple fruit* vs. *Apple company*. We did not observe a significant difference in noise for the top-10 and top-100 images. This is because categories such as *mango pulp* and *beef* have very few irrelevant images among the top-100 images, whereas *liquid rennin* has no relevant images overall. Hence, the number of relevant images remains almost the same for the top-10 and top-100 images. Nonetheless, we released all the 500 images to promote further research, such as visual queries using ingredient images and research related to tackling noise in the real-world. Sample ingredient images are included in the Supplementary material.

## 6. Conclusion and future research

To cluster similar recipe representations, we introduced a novel recipe similarity determinant that uses additional knowledge about recipes, such as titles and ingredients, while paying attention to rarely used ingredients. To incorporate this knowledge, we proposed a knowledge-infused learning network, Ki-Cook, to learn a multimodal cooking representation and cluster similar recipes in the latent space. Our experimental results demonstrated that clustering recipes through our similarity determinant retrieved relevant ingredients for an unknown food image compared to the base models. We also performed a qualitative analysis to illustrate the importance of rarely used ingredients in determining similar recipes to cluster them. We modeled the procedural attribute of the cooking process and incorporated a visual representation of ingredients to learn the multimodal cooking representation. For this purpose, we also extended the Recipe1M (Salvador et al., 2017) dataset with ingredient images constituting 8 million ingredient images in total and released the dataset to promote further research. Furthermore, our results demonstrated that infusing the knowledge and using the same deep learning models used in the base model (Salvador et al., 2017) can improve the results of ingredient retrieval for an unknown food image. In the future, we plan to include cooking methods in our similarity determinant and evaluate it for other downstream tasks such as predicting cooking methods, generating recipes, and meal recommendations.

## Data availability statement

The original contributions presented in the study are included in the article/Supplementary material, further inquiries can be directed to the corresponding author.

## Author contributions

RV, SP, and AS conceptualized and formalized the problem. RV, SR, RK, and ASR conducted the experiments and evaluations. AS supervised the project. All authors contributed to the writing of the paper. All authors contributed to the article and approved the submitted version.

## Footnotes

^1^Text representation refers to the learned representation generated for cooking instructions and ingredients of a given food image.

^2^Ground truth representation refers to the corresponding text representation of a food image representation and vice versa.

^3^Recipes collectively refers to the text representation and food image representation.

## Author disclaimer

Any opinions, findings, and conclusions or recommendations expressed in this material are those of the authors and do not necessarily reflect the views of the National Science Foundation. The food images used in the manuscript and the Supplementary material are collected from Google Images and is part of the dataset to be released for research purposes. The images were used under “The Fair Use provisions of the Copyright Act” which allows to use the copyrighted material for specific purposes including criticism, comment, news reporting, teaching, scholarship or research.
