# Supplementary material for: Ki-Cook: clustering multimodal cooking representations through knowledge-infused learning
Source: Front Big Data. 2023 Jul 24;6:1200840. doi: 10.3389/fdata.2023.1200840 (PMC10406211; doi:10.3389/fdata.2023.1200840)
Supplement: Supplementary file 1 [file Data_Sheet_1.PDF]

Supplementary Material

1 SUPPLEMENTARY IMAGES FOR QUALITATIVE ANALYSIS

In this section we present the retrieved ingredient images for the examples presented in Table 4. The retrieved images can be found in the Figures S1, S2 and S3

2 INGREDIENT ANALYSIS

We assess the quality of ingredient images as described in Section 4.4. The sample images are presented in Figure S4.

2.1 Figures

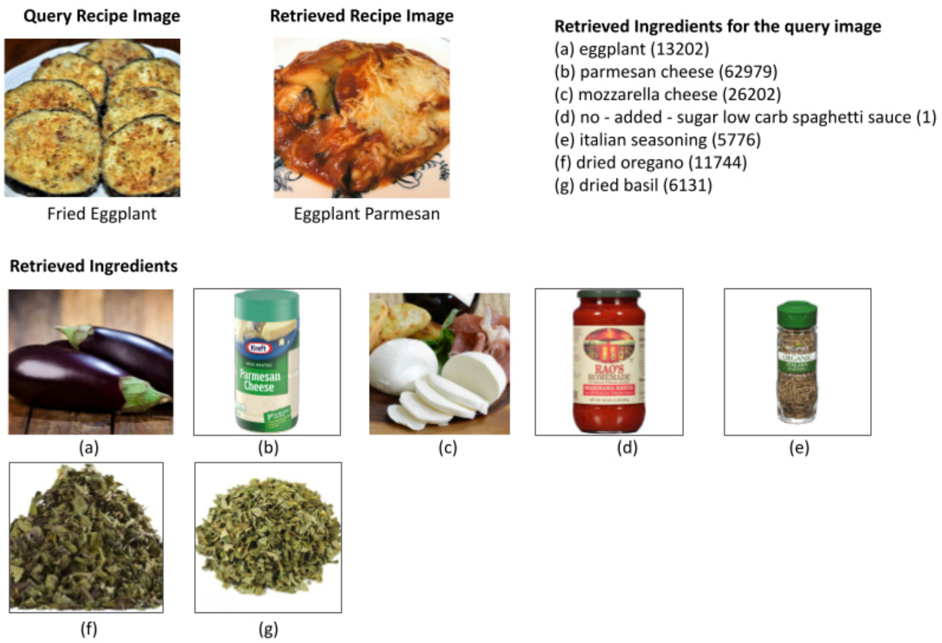

Figure S1. Retrieved ingredient images for the example-1 presented in Table 4

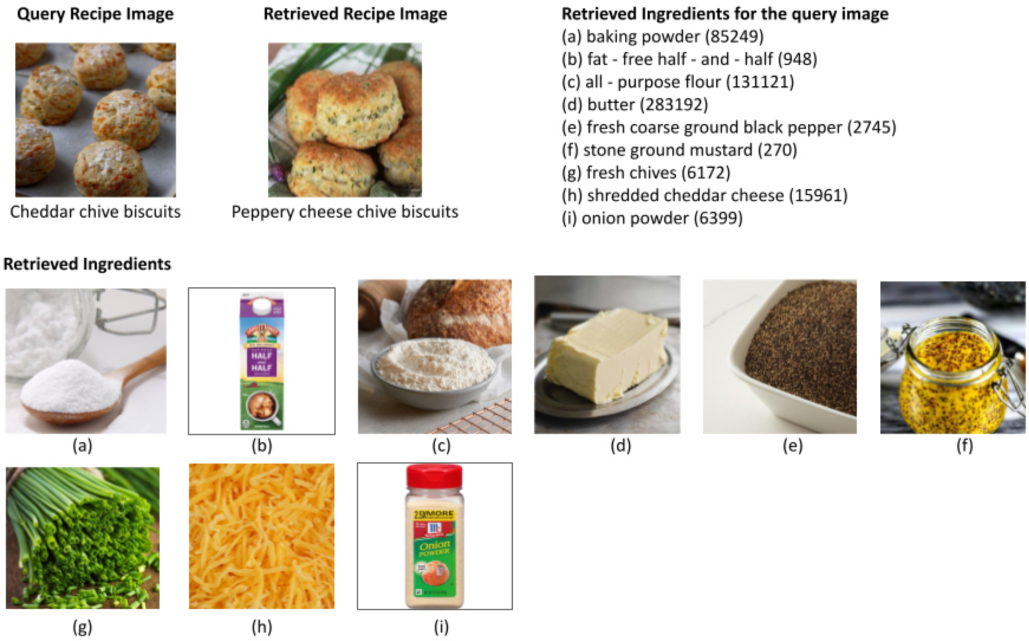

Figure S2. Retrieved ingredient images for the example-2 presented in Table 4

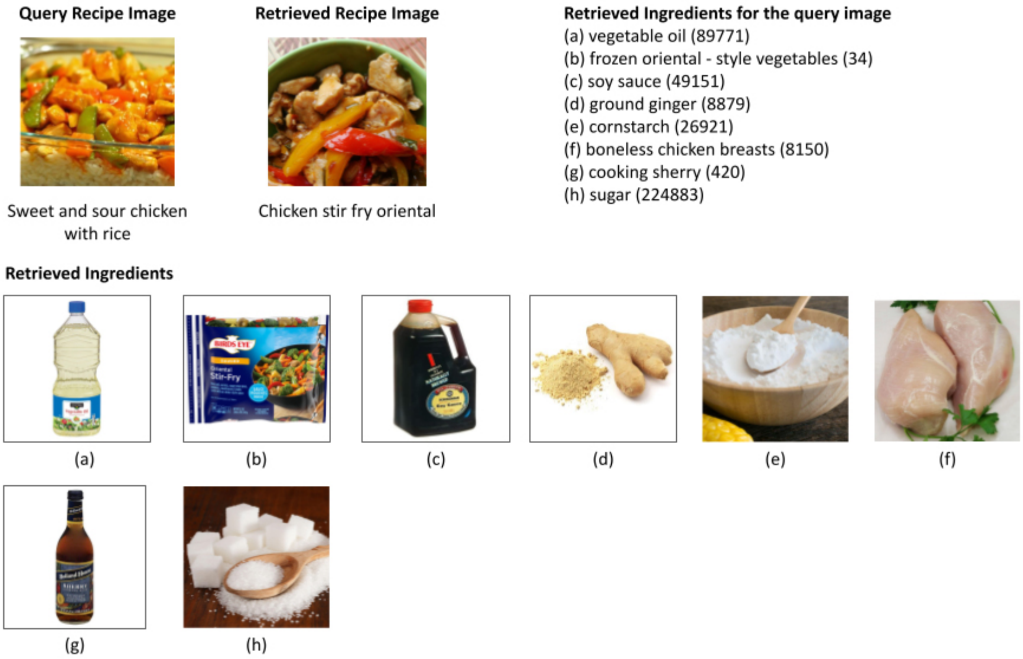

Figure S3. Retrieved ingredient images for the example-3 presented in Table 4

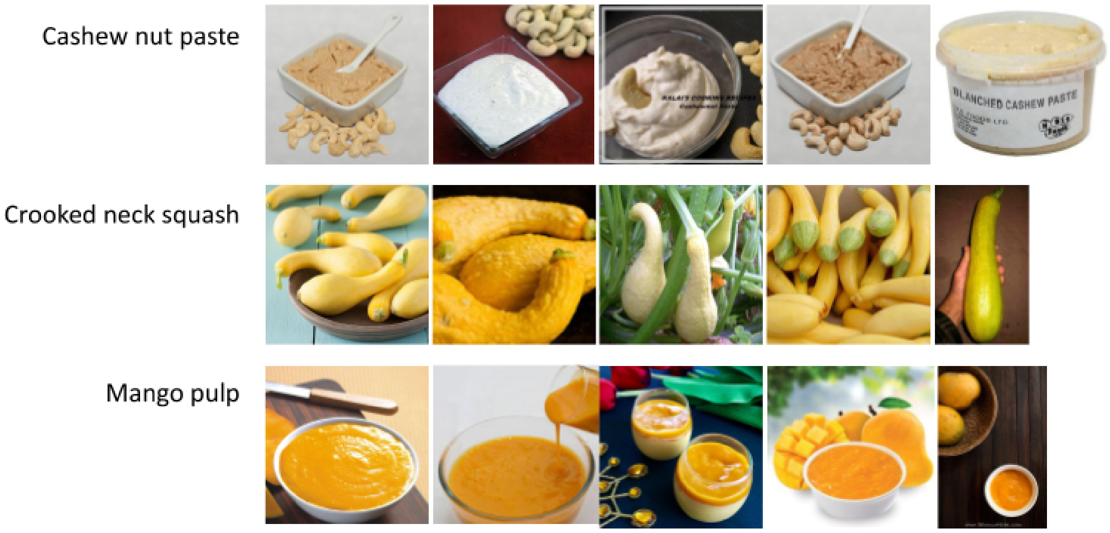

Figure S4. Sample of ingredient images collected using Google Images
